# Effect of Mindfulness-Based Social–Emotional Learning Approach on Knowledge and Anxiety Levels Regarding Cervical Cancer Prevention

**DOI:** 10.3390/healthcare14172684

**Published:** 2026-08-24

**Authors:** Nehal Shalaby Awad Mahmoud, Nourhan Essam Hendawi, Mahmoud Abdelwahab Khedr, Wafa Hamad Almegewly, Mazen Baazeem, Marwa Salah Abd Elgawad Abd Elhady

**Affiliations:** 1 Obstetrics and Gynecology Nursing Department, Faculty of Nursing, Alexandria University, Alexandria 21527, Egypt; nehal.shalaby@alexu.edu.eg (N.S.A.M.); marwa-salah@alexu.edu.eg (M.S.A.E.A.E.); 2 Nursing Education Department, Faculty of Nursing, Alexandria University, Alexandria 21527, Egypt; nourhan-assam@alexu.edu.eg; 3 Psychiatric and Mental Health Nursing Department, Faculty of Nursing, Alexandria University, Alexandria 21527, Egypt; 4 Department of Community and Psychiatric Mental Health Nursing, College of Nursing, Princess Nourah bint Abdulrahman University, P.O. Box 84428, Riyadh 11671, Saudi Arabia; whalmegewly@pnu.edu.sa; 5 Department of Health Information Management and Technology, College of Applied Medical Sciences, University of Hafr Albatin, Hafr Albatin 31991, Saudi Arabia; mbaazeem@uhb.edu.sa

**Keywords:** mindfulness-based social–emotional learning approach, knowledge, anxiety levels, cervical cancer prevention

## Abstract

**Background:** Cervical cancer remains a major public health concern worldwide, particularly in low- and middle-income countries. Limited knowledge regarding cervical cancer prevention and elevated anxiety levels may hinder women’s participation in preventive measures. Mindfulness-Based Social–Emotional Learning integrates mindfulness practices with social–emotional learning strategies and may enhance health knowledge while reducing anxiety. **Materials and Methods:** A quasi-experimental pretest–posttest non-equivalent control group design was conducted among 80 women attending gynecological outpatient clinics in Egypt. Participants were assigned to a study group (n = 40) and a control group (n = 40). Data were collected using a structured interview questionnaire, a cervical cancer knowledge questionnaire, and the Beck Anxiety Inventory (BAI). The study group received an eight-week MBSEL intervention incorporating mindfulness exercises, social–emotional learning activities, and cervical cancer education, while the control group received routine health education. Pre- and post-intervention assessments were performed. **Results:** Baseline characteristics were comparable between groups. Following the intervention, the study group had significantly higher mean knowledge scores regarding cervical cancer prevention, with mean knowledge scores increasing from 38.71 ± 10.41 to 66.25 ± 8.95 compared with 42.09 ± 15.95 in the control group (t = 8.355, *p* < 0.001). Good knowledge levels increased from 2.5% to 35.0%, while poor knowledge declined from 62.5% to 0.0%. Anxiety levels significantly improved in the study group, with mean BAI scores decreasing from 38.68 ± 6.32 to 22.20 ± 3.91 compared with 34.28 ± 9.53 in the control group (t = 7.414, *p* < 0.001). Low anxiety increased from 2.5% to 62.5% in the study group compared with 7.5% to 12.5% in the control group, and severe anxiety was eliminated from 27.5% to 0.0% in the study group compared with 32.5% to 22.5% in the control group after the intervention. **Conclusions:** The MBSEL approach effectively enhanced women’s knowledge regarding cervical cancer prevention and significantly reduced anxiety levels. Integrating mindfulness practices with social–emotional learning and health education appears to strengthen cognitive engagement and emotional regulation. **Clinical Trial Registration:** Registered in accordance with WHO and ICMJE standards (Trial number: PACTR202605512267205). Registration date: 29 May 2026.

## 1. Introduction

Cervical cancer is considered one of the most prevalent gynecological malignancies worldwide. It is characterized by a neoplasm that arises from the epithelial cells of the cervix, which is the inferior segment of the uterus that connects to the vagina. The progression of cervical cancer is generally insidious, initiating with precancerous alterations referred to as cervical intraepithelial neoplasia, which, if not identified and treated on time, may evolve into invasive carcinoma [1,2,3].

Globally, cervical cancer is the eighth most frequently diagnosed cancer and the fourth most common cancer among women, with an estimated 660,000 new cases reported each year and approximately 350,000 deaths annually [2,4,5]. In Egypt, around 1320 women receive a diagnosis of cervical cancer each year, with 744 fatalities resulting from the disease in 2021 [6].

The primary risk factor associated with cervical cancer is the persistent infection caused by high-risk variants of the human papillomavirus (HPV). Additional risk determinants encompass an early onset of initial sexual intercourse, engagement with multiple sexual partners, smoking, a compromised immune response (for instance, due to HIV/AIDS), and a documented history of cervical dysplasia or neoplasia. Furthermore, socioeconomic variables, the absence of routine Pap smear screenings, and restricted access to healthcare services may exacerbate the risk of developing cervical cancer. A comprehensive understanding of these risk factors is instrumental in enabling individuals to implement preventive strategies and pursue consistent screening, thereby reducing their susceptibility to the disease [2,7,8,9].

Nurses play a crucial role in enhancing public awareness concerning cervical cancer and associated preventive measures. The prevention or early detection of cervical cancer can be achieved through consistent screening practices, including Pap smears and HPV testing. The administration of the human papillomavirus (HPV) vaccine serves as a significant protective measure against the majority of cervical cancer cases by offering defense against high-risk HPV strains. Engaging in safe sexual practices, such as condom utilization and the limitation of sexual partners, contributes to a decreased likelihood of HPV infection. Additionally, the cessation of smoking and the adoption of a healthy lifestyle are instrumental in mitigating risk factors. Routine gynecological examinations and adherence to prescribed screening protocols are imperative for the timely detection and prevention of cervical cancer. By implementing these strategies, individuals can substantially diminish their probability of developing the malignancy [7,10,11].

Cervical cancer prevention is sometimes associated with anxiety, particularly concerning screenings and HPV testing. The apprehension surrounding the possibility of abnormal findings or the social stigma associated with HPV may induce anxiety. Nevertheless, regular screenings and HPV vaccinations are of paramount importance for prophylaxis. Addressing anxiety related to cervical health may necessitate the acquisition of knowledge regarding the risks and advantages of screenings, an understanding that early detection significantly enhances outcomes, and fostering transparent communication with healthcare professionals. By prioritizing proactive measures and support systems, individuals can alleviate anxiety and assert control over their cervical health, ultimately diminishing the likelihood of developing cervical cancer [12].

Mindfulness-Based Social–Emotional Learning (MBSEL) is a non-pharmacological educational approach that integrates mindfulness practices with social–emotional learning (SEL) competencies to promote emotional awareness, self-regulation, interpersonal skills, and adaptive decision-making. Mindfulness practices may include focused breathing, body scanning, and brief meditation, whereas SEL emphasizes competencies such as self-awareness, self-management, social awareness, relationship skills, and responsible decision-making [13,14]. Through the integration of these components, MBSEL may provide individuals with psychological and cognitive skills that support healthier responses to stressful situations and improve their capacity to engage with health-related information.

This approach presents a multitude of benefits, encompassing the management of stress, anxiety, and conflict, and fostering a positive and supportive educational environment. Through mindfulness-informed SEL, individuals are taught to identify and regulate their emotions, foster healthy interpersonal relationships, and cultivate essential life skills, including effective communication and problem-solving abilities, which collectively contribute to enhanced academic achievement, mental health, and overall success [15,16,17].

By integrating mindfulness within the SEL framework, individuals can develop a self-concept and navigate social and emotional challenges with greater efficacy. Ultimately, this approach bridges the gap between cognitive understanding and emotional readiness, supporting comprehensive preventive strategies and promoting holistic health outcomes among at-risk populations [15,17].

Several recent studies have reported that mindfulness-based social–emotional learning (SEL) interventions are effective in enhancing knowledge while reducing anxiety levels. For instance, [18,19,20,21] demonstrated that integrating mindfulness with SEL strategies improves emotional regulation, increases awareness and understanding of health-related information, and alleviates anxiety. Collectively, these findings highlight the value of mindfulness-based SEL approaches as an effective framework for promoting psychological well-being and strengthening knowledge among women.

Significance of the study: Cervical cancer is a predominant cause of mortality among women in low- and middle-income nations. This situation underscores considerable inequities driven by insufficient access to national HPV vaccination programs, cervical screening, treatment facilities, and the influence of social and economic determinants. Women living with HIV exhibit a sixfold increase in the likelihood of developing cervical cancer compared to the general population, with an estimated 5% of all cervical cancer incidences attributable to HIV. Cervical cancer disproportionately impacts younger women, and as a result, 20% of children who lose their mother to cancer do so due to cervical cancer [22,23].

There was little research in Egypt that addressed the effect of a mindfulness-based social–emotional learning approach on cervical cancer prevention. Consequently, the current study was initiated to examine the effect of a mindfulness-based social–emotional learning approach on knowledge and anxiety levels regarding cervical cancer prevention.

### Hypotheses

To reach the aim of this study, the following hypotheses were formulated:

**Hypothesis** **H1.**  *Women who follow the mindfulness-based Social–Emotional learning approach will exhibit a higher knowledge level than those who do not.*

**Hypothesis** **H2.**  *Women who follow the mindfulness-based Social–Emotional learning approach will exhibit lower anxiety levels than those who do not.*

Operational definition:

Mindfulness-based Social–Emotional learning approach: In this study, this approach consisted of mindfulness exercises, Social–Emotional Learning skills training, and educational sessions on cervical cancer prevention.

## 2. Methods

### 2.1. Research Design

A quasi-experimental design (pretest-posttest non-equivalent control group design) was adopted in the present study. A quasi-experimental design can be delineated as a research framework that bears resemblances to experimental research, although it does not fulfill the stringent criteria requisite for classification as true experimental research. In the pretest-posttest framework, the two dependent variables, knowledge and anxiety levels, were evaluated before and after implementation of the mindfulness-based social–emotional learning intervention [24]. The study was registered in accordance with WHO and ICMJE standards (Trial no: PACTR202605512267205, Registration date: 29 May 2026). The study was registered retrospectively because it was initially considered a quasi-experimental educational intervention. All study outcomes and planned assessments were pre-specified before participant enrollment, and no changes were made to the study protocol, outcomes, or planned assessments after enrollment.

### 2.2. Settings

This study was conducted at the Gynecological Outpatient Clinics of El-Shatby Maternity University Hospital, located in Alexandria Governorate, Egypt. The hospital provides free obstetric and gynecological healthcare services to women from Alexandria Governorate and surrounding areas through a multidisciplinary team of obstetricians and nurses. This setting was selected because it is a major university hospital providing comprehensive gynecological services and offering regular contact with women seeking healthcare. The availability of an appropriate clinical environment and opportunities for interaction with women made this setting suitable for implementing the Mindfulness-Based Social–Emotional Learning (MBSEL) intervention, including its educational and psychological components related to cervical cancer prevention.

### 2.3. Subjects

A convenience sample of 80 women was recruited for this study. Women who met the eligibility criteria and agreed to participate were enrolled after receiving a clear explanation of the study objectives and procedures. The inclusion criteria were women aged 20–50 years, married, literate, and willing to participate in the study. Literacy was assessed based on participants’ ability to independently read and complete the study questionnaires. Married women were included because they represent the predominant group attending gynecological outpatient clinics and are more likely to utilize reproductive health services related to cervical cancer prevention. Women were excluded if they had a history of cervical cancer, a current diagnosis of cervical cancer, a previous hysterectomy, or any prior cervical surgical intervention.

**Figure 1:**
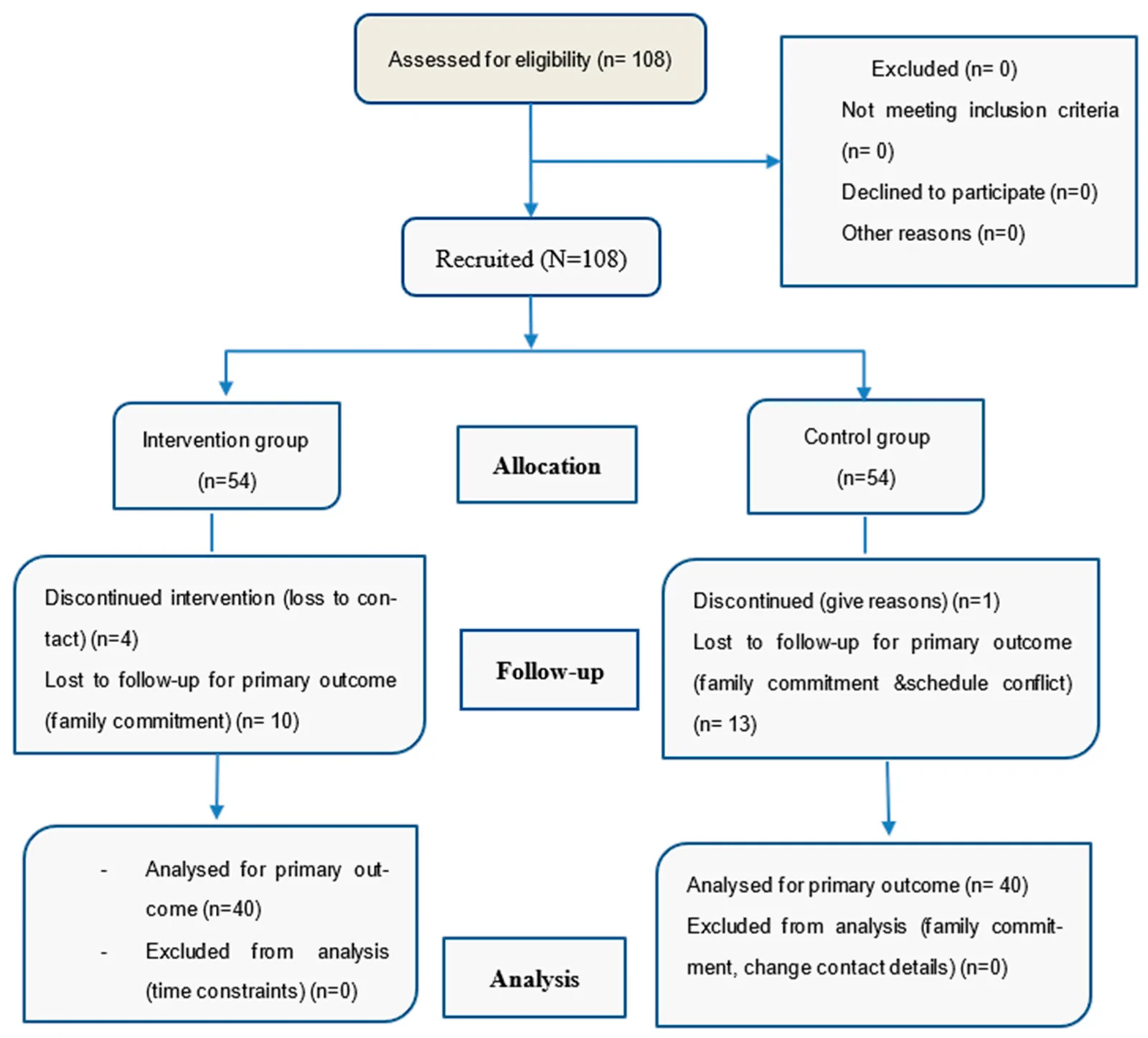
TREND Flow Diagram.

Sample size estimation was performed a priori using G*Power (Version 3.1) employing an ANCOVA model with fixed effects, main effects, and interactions within an F-test family involving two groups and one covariant. Using a large effect size (f = 0.4) based on Cohen’s guidelines, a level of significance of α = 0.05, and a statistical power of 90% [25]. The minimum required sample size was estimated as n = 68 participants. However, to account for possible non-response and attrition during the follow-up period of the study, the sample size was increased to 108 participants. Out of 108 participants recruited at the beginning of the study (54 for each group), 28 were either discontinued or lost to follow-up from both groups, ending with a final sample of 80 participants (40 participants per group); a detailed participant recruitment flow diagram was presented in Figure 1.

### 2.4. Study Tools

Three tools were employed for data collection.

Tool (I): Structured interview questionnaire: This tool was developed and implemented by the researcher following a comprehensive review of contemporary and pertinent literature to gather the subsequent data. It comprised four sections: the initial section included demographic data encompassing age, educational level, occupational status, family type, residence, and income. The second section addressed menstrual history, which encompassed age at menarche, menstrual regularity, interval, duration, amount of blood loss (measured in number of pads per day), associated dysmenorrhea, and abnormal bleeding during non-menstrual days. The third section focused on reproductive history, which comprised gravidity, parity, history of sexually transmitted diseases, history of infections within the reproductive system, contraceptive methods employed, and familial history of cervical cancer.

Tool (II): Knowledge about cervical cancer questionnaire: This tool was utilized to evaluate women’s knowledge regarding cervical cancer. It was adapted from Harsha Kumar and Tanya (2014) [26] and further modified by the researchers to suit the objectives of the current study. The questionnaire consisted of 18 items covering various aspects of cervical cancer, including its definition, associated risk factors, etiological factors, signs and symptoms, diagnostic procedures, progression, treatment modalities, side effects of chemotherapy and radiotherapy, preventive measures, vaccination protocols, Pap smear screening, appropriate screening settings, and possible complications. All items were designed in the form of multiple-choice questions (Supplementary Table S1).

Regarding the scoring system, each correct response was assigned a score of one, while incorrect responses received a score of zero. The scores of all items were summed to obtain the total knowledge score, with the maximum possible score being 18. The obtained scores were then converted into percentage scores to facilitate interpretation. Knowledge levels were classified into three categories: poor knowledge (≤33%), fair knowledge (34% to less than 70%), and good knowledge (70% and above). For use among Egyptian women, the questionnaire was translated into Arabic and culturally adapted by the researchers. The translation and adaptation process included careful review of the wording to ensure semantic clarity, cultural appropriateness, and comprehensibility within the Egyptian context. The Arabic version was subsequently reviewed by a panel of three experts specializing in obstetric and gynecological nursing and psychiatric nursing to assess the relevance, clarity, semantic appropriateness, and cultural suitability of the items. The internal consistency of the final questionnaire was assessed using Cronbach’s alpha in the main study sample and demonstrated satisfactory reliability (Cronbach’s α = 0.876).

Tool (III): Beck Anxiety Inventory (BAI): This scale was adapted and utilized by the researchers to assess anxiety levels among women. The Beck Anxiety Inventory (BAI), developed by Beck et al. (1988) [27], is a self-report assessment tool designed to measure the severity of anxiety symptoms. The scale consists of 21 items that assess emotional, cognitive, and physical symptoms experienced during the preceding four weeks. These symptoms include numbness or tingling sensations, feelings of heat, inability to relax, fear of the worst happening, dizziness or light-headedness, rapid heartbeat, shakiness, and unsteadiness.

The scale was measured using a four-point Likert response format ranging from 0 to 3, where 0 indicated “not at all,” 1 indicated “mildly but not significantly bothersome,” 2 indicated “moderately, it was somewhat unpleasant,” and 3 indicated “severely, it was highly bothersome.” The total score ranged from 0 to 63, with higher scores indicating greater anxiety severity. Anxiety levels were categorized into three levels: low anxiety (0–21), moderate anxiety (22–35), and severe anxiety (36 and above), consistent with the categorization used in previous studies [28,29]. The Arabic version of the BAI has been previously evaluated among Arabic-speaking populations and demonstrated acceptable validity and reliability, supporting its use for assessing anxiety symptoms in Arab populations. Previous research has examined the applicability of the BAI among Arab college students and reported evidence supporting its use in this cultural context [28,30]. The internal consistency of the final questionnaire was evaluated using Cronbach’s alpha, yielding a satisfactory reliability coefficient of 0.883.

### 2.5. Validity and Reliability of the Tools

The tools underwent content validity testing conducted by a jury of three experts specializing in obstetric, gynecologic, and psychiatric nursing, leading to the implementation of necessary modifications. The internal consistency reliability of the study instruments was assessed using data obtained from the full sample (n = 80) by calculating Cronbach’s alpha coefficients. Tools II and III demonstrated satisfactory reliability, with Cronbach’s alpha values of 0.876 and 0.883, respectively.

### 2.6. Ethical Consideration

The study was conducted in accordance with the principles of the Declaration of Helsinki. Official permission was obtained from the Research Ethics Committee at the Faculty of Nursing, Alexandria University, to validate the study tools and conduct the research (Research code: Au-20-3-414, dated 15 October 2025). Written informed consent was obtained from all participants before data collection. After providing a comprehensive explanation of the study objectives and procedures, participants were assured of their privacy, confidentiality, voluntary participation, and right to withdraw at any time without any consequences. To maintain anonymity, participants’ names were not recorded on the study questionnaires; instead, unique identification codes were used. All collected data were securely stored in password-protected electronic files and kept in a secure location accessible only to the research team. Regarding the WhatsApp group used as part of the intervention, participants were informed about the purpose of the group and provided consent before joining. They were advised not to share personal health information within the group, and all shared materials and communications were handled confidentially and used solely for study purposes.

### 2.7. Pilot Study

A pilot study was conducted involving 10% of the study sample (8 women), who were subsequently excluded from the main study sample. The pilot study was conducted to assess the applicability and clarity of the assessment tools and to estimate the time required for data collection. Based on the findings of the pilot study, no major modifications were required; however, minor clarifications were made to ensure the items were clear and understandable to participants before the main study.

### 2.8. Study Procedure

The study was conducted according to the following phases:

#### 2.8.1. Preparatory Phase

Tool (I) was developed by the researcher following a thorough review of current and pertinent literature. Tools (II) and (III) were adapted and translated into Arabic for the purposes of data collection. Then approval was secured from the vice-dean of graduate studies & research at the Faculty of Nursing, Alexandria University, and was submitted to the relevant authorities overseeing the study settings to obtain their consent for data collection, accompanied by a detailed explanation of the study’s objectives.

#### 2.8.2. Assessment Phase

At the beginning, the researchers engaged with women during their waiting periods at gynecological outpatient clinics. They presented their identity and elucidated the title and objectives of the study. Subsequently, the researchers conducted individual interviews with each woman to complete a structured interview questionnaire.

After that, the researchers assessed the women’s knowledge and anxiety regarding cervical cancer prevention (pretest) utilizing tools, a knowledge about cervical cancer questionnaire, and a Beck Anxiety Inventory. Each woman required approximately 20 to 30 min to complete all questionnaires, with some being filled out by the women themselves and others by the researchers, contingent upon each woman’s level of education. The researchers conducted visits to the aforementioned setting three days per week (Sunday, Tuesday, Thursday) from 9:00 a.m. to 2:00 p.m., in accordance with the outpatient clinic schedule of the study location.

#### 2.8.3. Planning Phase

The Mindfulness-Based Social–Emotional Learning (MBSEL) program was carefully developed based on evidence-based practices in mindfulness and social–emotional learning (SEL). The program was specifically designed to incorporate educational components related to cervical cancer prevention while addressing the psychological and emotional needs of women. Special attention was given to developing culturally sensitive content appropriate for the target population. The educational modules covered essential topics related to cervical cancer, including risk factors, the relationship between human papillomavirus (HPV) and cervical cancer, the importance of regular screening through Pap smear and HPV testing, and preventive measures such as HPV vaccination and healthy lifestyle modifications.

The program objectives were formulated to enhance women’s knowledge and emotional well-being. The general objective of the program was that, upon completion of the mindfulness-based social–emotional learning sessions, women would acquire adequate knowledge regarding cervical cancer and its prevention while also experiencing a reduction in anxiety levels. The specific objectives aimed to enable women to identify the risk factors, signs, and symptoms associated with cervical cancer, recognize possible complications of the disease, understand preventive measures, adopt healthy preventive behaviors, and correct misconceptions and misunderstandings related to cervical cancer and its screening procedures.

Before implementation, the finalized MBSEL program underwent a comprehensive review and evaluation by a panel of academic and clinical experts in the fields of obstetric and gynecological nursing and mental health to ensure the clarity, relevance, cultural appropriateness, and scientific accuracy of the program content.

#### 2.8.4. Implementation Phase

Data collection was carried out over a period of eight months, extending from the beginning of November 2025 to the end of June 2026. The recruited women were allocated into two main groups. The control group consisted of 40 women who received routine health education sessions during the same study period. The sessions covered general health topics unrelated to cervical cancer prevention or mindfulness practices, including healthy nutrition, physical activity, and general lifestyle recommendations. The health education was delivered through structured group sessions using standard educational materials. It was provided for the same duration and frequency as the study group to ensure comparable contact time. The sessions did not include any psychological, mindfulness-based, or social–emotional learning components. The study group included 40 women who received the Mindfulness-Based Social–Emotional Learning (MBSEL) intervention.

1. Scheduling

The implementation phase of the MBSEL intervention included scheduling, session delivery, and strategies to enhance women’s engagement. The intervention was conducted over eight weeks, including one week for orientation and pretesting, six weeks for intervention implementation, and one week for post-testing and feedback. Both the intervention and control groups attended one session weekly throughout the study period. Sessions were conducted in a designated community room within the collaborating health centers to provide a quiet, comfortable, and supportive environment. To ensure intervention fidelity, a standardized protocol was developed and followed throughout the implementation of the MBSEL program. Each session was delivered according to predefined objectives, content, and activities. The researcher used a structured checklist to monitor the delivery of the intervention components and ensure consistency across sessions. Regular review of session implementation was performed to confirm adherence to the intervention protocol.

2. Delivery of Intervention Sessions

Each intervention session was conducted weekly by trained researchers and lasted approximately 30 min. Women were divided into small groups of five to six participants to encourage interaction, emotional support, and active participation. Each MBSEL session followed a structured format. Sessions began with a welcome and emotional check-in, during which women were encouraged to express their current emotional states through brief guided questions, such as “How are you feeling today?” This process promoted emotional openness and strengthened group cohesion.

The mindfulness component included breathing exercises, guided imagery, body scan exercises, and relaxation techniques. Audio recordings and standardized scripts were used to maintain consistency across sessions. The social–emotional learning component addressed topics such as self-awareness, emotional regulation, empathy, interpersonal communication, and decision-making skills. These topics were delivered through interactive lectures, group activities such as role play and emotion mapping, and peer discussions designed to encourage engagement and reflection.

The cervical cancer awareness component focused on increasing women’s understanding of cervical cancer prevention. Interactive educational discussions addressed topics such as HPV infection, signs and symptoms of cervical cancer, screening methods, preventive measures, HPV vaccination, and healthy lifestyle practices. Educational strategies included the use of visual aids, posters, anatomical diagrams, multimedia presentations, short video clips, and weekly informational handouts containing infographics. In addition, recorded survivor testimonials were presented during selected sessions to enhance emotional connection and awareness.

At the end of each session, women participated in reflection and group discussion activities, during which they shared their thoughts, feelings, and questions related to the session content. The MBSEL intervention was systematically organized over eight successive weeks, with each session integrating mindfulness practices, social–emotional learning skills, and cervical cancer education to provide a comprehensive and cohesive learning experience (Table 1).

3. Women’s Engagement and Reinforcement

To enhance participant engagement and reinforce the intervention, several supportive strategies were implemented throughout the program. A WhatsApp support group was established to facilitate continuous communication with participants. Through this platform, daily reminders, motivational messages, and summaries of mindfulness techniques were regularly shared to encourage adherence to the intervention and maintain women’s engagement throughout the study period.

In addition, each woman received a printed Arabic booklet designed for home practice. The booklet included guidance on mindfulness exercises and provided space for participants to document their daily mindfulness practices, including the duration, type of activity performed, and their emotional responses following each exercise. This strategy aimed to strengthen self-monitoring, promote regular practice, and enhance the integration of mindfulness techniques into daily life.

To further encourage participation and create a supportive atmosphere, healthy snacks were provided during the intervention sessions. Moreover, women who completed the program received complimentary cervical cancer prevention vouchers and certificates of participation during the final session as a form of appreciation and motivation.

#### 2.8.5. Evaluation Phase

The researcher reevaluated the knowledge and anxiety levels toward cervical cancer and its prevention (posttest) of both groups following three months of mindfulness-based social–emotional learning implementation by using tools (II) and (III). The effect of mindfulness-based social–emotional learning on knowledge and anxiety levels was determined by comparing the mean knowledge and anxiety levels after the implementation.

### 2.9. Statistical Analysis

Data were input into the computer and analyzed utilizing the IBM SPSS software package version 26.0. Qualitative data were characterized using numerical values and percentages, as well as mean and standard deviation. The significance of the results obtained was assessed at the 5% level. The methodologies employed included the chi-square test for categorical variables to compare disparate groups, with Monte Carlo correction applied for chi-square when more than 20% of the cells exhibited expected counts of less than 5. The student t-test was utilized for normally distributed quantitative variables to compare the two studied groups. A linear mixed-effects model (LMM) was used to assess the effects of Group, Time, and the Group × Time interaction on the outcome measures. In addition, ANCOVA was used to compare post-intervention outcomes between the study groups while adjusting for baseline values. The Δ (change score) was calculated as the difference between the post-intervention and pre-intervention scores.

## 3. Results

Table 2: Elaborates on demographic attributes of the study participants. The mean age was 34.93 ± 5.34 and 36.48 ± 4.90 years for both study and control groups, respectively. As regards educational attainment, a sizable proportion (45%, 52.5%) of both groups had secondary education, respectively. In relation to occupation, (70%) of the study group, compared to (72.5%) of the control group, were housewives. Regarding current residence, more than half (55%) of the study group versus less than half (45%) of the control group were rural indwellers. For economic status, almost three-quarters (75%, 72.5%, respectively) of them had enough income. In relation to the type of family, more than half (55%, 52.5%, respectively) of the two groups had an extended family. No statistically significant differences were found between groups regarding demographic characteristics.

Table 3: Demonstrates the distribution of the study participants according to their menstrual history. No statistically significant differences were found between groups regarding their menstrual history. Where less than half (47.5%) of the study group, compared to about two-thirds (65%) of the control group, had menstruation before 14 years old. While (65%, 72.5%, respectively) of the two groups had regular menstrual cycles. Meanwhile, (72.5%, 77.5%) of both the study and control groups, respectively, had a 21–35-day interval between menstruation. The majority (82.5%) of the study group, compared to approximately three-quarters (72.5%) of the control group, had menstruation lasting for 3–7 days. Slightly more than two-thirds (67.5%) of the study group versus 75% of the control group had pain during menstruation. The minority (7.5%, 15.0%, respectively) of both the study and control groups had bleeding during menstruation.

Table 4: Demonstrates the distribution of the study participants according to their reproductive background. The majority (85%) of the study group versus the vast majority (92.5%) of the control group were multigravida and multipara, respectively. At the same time, 95% and 97.5% of the two groups had no previous history of sexually transmitted disease. Meanwhile, 57.5% of the study group, compared to half (50%) of the control group, had no previous history of genital tract infection. Regarding contraceptive use, 95% of the study group versus 85% of the control group had used contraceptive methods, with oral contraceptive pills and IUDs being the most commonly used. The vast majority (95%, 92.5%, respectively) of both groups had no family history of cervical cancer.

Table 5 clarifies comparison between both study and control groups based on their knowledge regarding cervical cancer. Before the intervention, more than three-fifths (62.5%) of the study group and less than half (47.5%) of the control group had a poor level of knowledge. But post-intervention, a statistically significant difference was observed between the intervention and control groups in the distribution of knowledge categories (*p* < 0.001). Notably, none of the participants in the intervention group were classified in the poor knowledge category. In contrast, the control group showed minimal change: 37.5% had a poor level, and only 5% remained at a good level after the same period. These distinct shifts in percentages highlight the significant impact of the intervention on knowledge acquisition.

Table 6 portrays comparison between the study and control groups according to their level of anxiety using the Beck Anxiety Inventory (BAI). Before the intervention, 2.5% of the study group, compared to 7.5% of the control group, had a low level of anxiety with no statistically significant difference between the two groups (*p* = 0.512). Meanwhile, after the intervention, a statistically significant difference was observed between the two groups in favor of the study group (*p* < 0.001), i.e., women in the study group were more likely to benefit from the used intervention, where (62.5%) of the study group had a low anxiety level, and none remained at a high level. Conversely, only 12.5% of the control group reached the low level. These changes in the percentages reflect the significant positive impact of the intervention on anxiety levels.

## 4. Discussion

Cervical cancer remains a major global health concern, with limited knowledge and high anxiety often hindering engagement in preventive measures. Mindfulness-based social–emotional learning can help overcome these barriers by enhancing self-awareness, supporting emotional regulation, and encouraging participation in early detection and health education [18,31]. This study examined the effect of mindfulness based social–emotional learning approach on knowledge and anxiety levels regarding cervical cancer prevention.

The study’s findings revealed that more than half of the women in both study and control groups were older than 35 years. Also, more than half of the study group and nearly half of the control group were rural indwellers. These findings are consistent with Shawky, Mohammed [32], who reported that the studied women were aged between 30 and 39 years, with most residing in rural areas. Similarly, Romli, Shahabudin [33] observed that most women were between 35 and 49 years old. Moreover, Debnath, Pal [34] found that the majority of participants lived in rural settings.

Moreover, slightly less than half of the study group, compared to nearly two-thirds of the control group, experienced menarche before the age of fourteen. In addition, more than half of the women in both groups reported having a regular menstrual cycle. These findings are consistent with Okyere, Ayebeng [35], who reported that early menarche significantly influences women’s engagement in cervical cancer screening behaviors. Similarly, Crespo, Herranz [36] found that the average age at menarche among participants was approximately 13 years, and the majority reported regular menstrual cycles.

Furthermore, a notable proportion of women in both groups reported a history of vaginal infections and the use of contraceptive methods, with oral contraceptive pills and intrauterine devices (IUDs) being the most used. At the same time, the majority of women did not report a family history of cervical cancer. These findings are supported by Okyere, Ayebeng [35], who demonstrated that a history of sexually transmitted infections significantly influences the likelihood of cervical cancer screening uptake. Similarly, Fawzy, Hossien [37] reported that women with a history of infections and those using oral contraceptive pills exhibited particular reproductive health patterns relevant to cervical cancer risk and preventive behaviors. Moreover, Osman, Keshk [38] reported that most women identified a family history of the disease as an influencing factor for screening behavior. In contrast, Ikegwuonu, Ewenyi [39] found that most participants had never heard of cervical cancer.

Regarding the first hypothesis—women who follow the mindfulness-based social–emotional learning approach will exhibit a higher knowledge score than those who do not—in the current study, there was a statistically significant improvement in knowledge levels post-intervention, favoring the study group. The substantial improvement in knowledge may be explained by the complementary contribution of the three MBSEL components. The educational component may have contributed most directly to knowledge acquisition by providing structured information on cervical cancer prevention, while mindfulness may have enhanced attention and engagement with the learning content. Similarly, social–emotional learning may have supported emotional and learning readiness, thereby facilitating engagement with educational materials. Thus, the pronounced knowledge gain may reflect the integrated effect of structured health education supported by mindfulness and social–emotional learning rather than mindfulness alone. Although the individual effects of these components were not isolated, the educational component may have contributed more direct to knowledge acquisition, with mindfulness and social–emotional learning acting as complementary facilitators of the learning process [40,41].

This finding is consistent with results reported in several other studies. Qin and Liu [18] examined mindfulness interventions among post–cervical cancer women and found that these interventions significantly improved health knowledge and satisfaction. In addition, Inbar and Tarrasch [42] observed that students who participated in mindfulness-based lessons showed better knowledge and achieved higher test scores than those in the control group.

Furthermore, Bordbar and Ahmadinejad [43] examined the impact of mindfulness on students’ academic achievement, highlighting the mediating role of adaptability. Their findings showed that mindfulness enhances academic performance by improving attention, cognitive flexibility, and information processing, which enables learners to focus more effectively on study materials and retain knowledge more efficiently. Moreover, Debnath and Pal [34] reported a notable increase in knowledge scores among women who received health education interventions compared with those in the control group.

As regards the second hypothesis—women who follow the mindfulness-based social–emotional learning approach will exhibit lower anxiety levels than those who do not—in the current study, there was a statistically significant reduction in anxiety levels in favor of the study group. This improvement can be attributed to the mindfulness-based social–emotional learning intervention, which enhances emotional regulation, promotes stress management, and increases self-awareness. By cultivating a state of calm focus, the intervention enabled women to manage concerns related to cervical cancer prevention more effectively, engage confidently with health education, and adopt proactive preventive behaviors [14,44]. This finding is in line with Qin and Liu [18], who reported that participants who received mindfulness-based cognitive behavioral interventions demonstrated significant improvements in physiological status, emotional well-being, and functional status compared to the control group.

Similarly, Kim and Kim [19] reported that a Mindfulness-Based Social–Emotional Growth Program significantly enhanced emotional regulation and reduced anxiety in the study group. Likewise, Alvarado-García and Soto-Vásquez [20] found that mindfulness interventions effectively reduced stress, anxiety, and depression while improving sleep quality, social support, and life satisfaction. Additionally, Li and Lei [21] demonstrated reductions in anxiety and improvements in mental health scores among participants receiving mindfulness-based interventions. Moreover, Bahri and Montazeri [45] observed that participants who completed eight mindfulness training sessions showed significant improvements in overall quality of life, psychological well-being, and reductions in anxiety.

On the other hand, D’Souza and Smyth [46] reported that no significant improvement in anxiety was observed in the study group compared to control conditions after mindfulness-based interventions. Similarly, Liu and Cai [47] found that although mindfulness-based interventions significantly reduced stress in menopausal women, there were no statistically significant effects on anxiety or depression.

The observed improvement in anxiety level in the present study may be attributed to the positive impact of mindfulness on learning processes and emotional regulation. In addition, integrating mindfulness into social–emotional learning enhances self-awareness, emotion regulation, and cognitive engagement, thereby positively influencing educational outcomes and knowledge development [48].

## 5. Conclusions

The results indicated that women who participated in the mindfulness-based social–emotional learning approach showed improved knowledge regarding cervical cancer prevention and reduced anxiety levels compared with the control group.

## 6. Recommendations

Based on the findings of the present study, mindfulness-based social–emotional learning approaches may be considered as a potential supportive strategy to enhance women’s health education and promote preventive healthcare practices. However, further evidence is needed before recommending widespread integration into maternity healthcare policies. Increasing awareness among healthcare professionals regarding mindfulness-based social–emotional learning approaches may facilitate future implementation in clinical and educational settings.

Furthermore, future research may explore the integration of mindfulness-based social–emotional learning approaches within nursing and healthcare education curricula to examine their potential benefits for learning engagement and knowledge retention. Additional studies are warranted to assess women’s satisfaction with such interventions and to evaluate healthcare professionals’ knowledge and practices regarding the application of mindfulness-based approaches.

Future studies using randomized controlled designs, larger and more diverse samples, multiple healthcare settings, and attention-matched control groups are recommended to minimize potential confounding variables and provide stronger evidence regarding the effectiveness of mindfulness-based social–emotional learning approaches in improving cervical cancer prevention knowledge and reducing anxiety levels.

Trial Registration: The study was registered in accordance with WHO and ICMJE standards (Trial no: PACTR202605512267205).

## 7. Limitations of the Present Study

This study has several limitations that should be acknowledged. First, the use of a convenience sample and a quasi-experimental design may limit the generalizability of the findings and introduce selection bias. Second, although the study protocol and outcome measures were finalized before participant enrollment and remained unchanged throughout the study, the trial was registered retrospectively because it was initially considered a quasi-experimental educational intervention. Third, contamination between the intervention and control groups could not be completely excluded, as participants were recruited from the same outpatient setting. In addition, blinding of participants and researchers was not feasible due to the educational nature of the MBSEL intervention, which may have introduced performance and assessment bias.

Furthermore, the relatively short follow-up period limited the evaluation of the long-term sustainability of the intervention effects. Also, the intervention incorporated several adherence-enhancing strategies, including WhatsApp support, daily reminders, home practice materials, and motivational incentives. These additional supportive elements may have increased participants’ engagement and perceived support, which could have influenced the study outcomes. Therefore, the independent contribution of the mindfulness-based social–emotional learning component cannot be completely isolated. Finally, knowledge and anxiety were assessed using self-reported measures, which may be subject to response and social desirability bias. Future studies employing randomized controlled designs, probability sampling, prospective trial registration, longer follow-up periods, and objective outcome measures are recommended to strengthen the evidence.

## Figures and Tables

**Table 1:** Mindfulness-Based Social–Emotional Learning Sessions.

Week	Theme	Mindfulness Practice	SEL Focus	Cervical Cancer Module
Week 1	Introduction & Grounding	Breathing awareness	Self-awareness, group norms	What is cervical cancer? Basic facts
Week 2	Emotional Recognition	Body scan meditation	Recognizing & naming emotions	Causes and risk factors
Week 3	Managing Stress & Anxiety	Guided imagery	Stress management techniques	HPV and its link to cervical cancer
Week 4	Compassion & Empathy	Loving-kindness meditation	Building empathy and compassion	Common myths and stigma
Week 5	Communication Skills	Mindful listening	Assertive communication	Pap smear: What to expect
Week 6	Decision Making	Anchoring breath	Responsible decision-making	HPV vaccine: Benefits and eligibility
Week 7	Resilience Building	Walking meditation	Coping with fear and uncertainty	Screening guidelines and timelines
Week 8	Reflection & Integration	Silent meditation	Integration of skills	Creating personal health action plans

**Table 2:** Distribution of the studied women according to their demographic attributes (N = 80).

Demographic Attributes	Study (n = 40)		Control (n = 40)		χ^2^	^MC^p
	Freq.	%	Freq.	%		
**Age**						
20–	4	10.0%	2	5.0%		
26–	5	12.5%	3	7.5%		
31–	7	17.5%	8	20.0%	1.439	0.697
>35	24	60.0%	27	67.5%		
Mean ± SD	34.93 ± 5.34		36.48 ± 4.90			
**Education**						
Read and write	1	2.5%	5	12.5%		
Primary education	9	22.5%	4	10.0%		
Secondary education	18	45.0%	21	52.5%	4.780	0.171
University education	12	30.0%	10	25.0%		
**Occupation**						
Housewife	28	70.0%	29	72.5%		
Employee	12	30.0%	11	27.5%	0.061	0.805
**Type of family**						
Nuclear	22	45.0%	21	47.5%		
Extended	18	55.0%	19	52.5%	0.050	0.823
**Residence**						
Urban	18	45.0%	22	55.0%		
Rural	22	55.0%	18	45.0%	0.800	0.371
**Income**						
More than enough	0	0.0%	0	0.0%		
Just enough	30	75.0%	29	72.5%	0.065	0.799
Not enough	10	25.0%	11	27.5%		

χ^2^: Chi-square test; MC: Monte Carlo t: Student *t*-test.

**Table 3:** Distribution of the studied women according to their menstrual history (N = 80).

Menstrual History	Study (n = 40)		Control (n = 40)		χ^2^	^MC^p
	Freq.	%	Freq.	%		
**Age of menarche**						
<14 years	19	47.5%	26	65.0%		
14–16 years	20	50.0%	14	35.0%	3.057	0.180
>16 years	1	2.5%	0	0.0%		
**Regularity**						
Yes	26	65.0%	29	72.5%	0.524	0.630
No	14	35.0%	11	27.5%		
**Interval of menstruation**						
<21 days	7	17.5%	2	5.0%		
21–35 days	29	72.5%	31	77.5%	3.530	0.153
>35 days	4	10.0%	7	17.5%		
**Duration of menstruation**						
<3 days	1	2.5%	5	12.5%		
3–7 days	33	82.5%	29	72.5%	2.788	0.284
>7 days	6	15.0%	6	15.0%		
**Number of pads per day**						
<3	16	40.0%	19	47.5%		
3–5	23	57.5%	20	50.0%	0.716	0.817
>5	1	2.5%	1	2.5%		
**Pain during menstruation**						
Yes	27	67.5%	30	75.0%	0.549	0.622
No	13	32.5%	10	25.0%		
**Bleeding during menstruation**						
Yes	3	7.5%	6	15.0%	1.127	0.288
No	37	92.5%	34	85.0%		

χ^2^: Chi-square test; MC: Monte Carlo t: Student *t*-test.

**Table 4:** Distribution of the studied women according to their reproductive history (N = 80).

Reproductive History	Study (n = 40)		Control (n = 40)		χ^2^	^MC^p
	Freq.	%	Freq.	%		
**Gravidity**						
Nulligravida	0	0.0%	0	0.0%		
Primigravida	5	12.5%	3	7.5%		
Multigravida	34	85.0%	37	92.5%	3.182	0.371
Grand multigravida	1	2.5%	0	0.0%		
**Parity**						
Nullipara	0	0.0%	0	0.0%		
Primipara	5	12.5%	3	7.5%		
Multipara	34	85.0%	37	92.5%	1.843	0.427
Grand multipara	1	2.5%	0	0.0%		
**Previous history of sexually transmitted diseases**						
No	38	95.0%	39	97.5%		
Yes	2	5.0%	1	2.5%	1.053	0.615
**If yes: use any method for protection**						
Yes	2	100%	1	100.%		
No	0	0.0%	0	0.0%	0.444	1.000
**Previous history of genital tract Infection**						
No	23	57.5%	20	50.0%		
Yes	17	42.5%	20	50.0%	0.453	0.501
**Contraceptive use**						
No	2	5.0%	6	15.0%		
Yes	38	95.0%	34	85.0%	2.222	0.263
**Type of contraceptive**	**N = 38**		**N = 34**			
pills	18	47.4%	14	41.2%		
Natural FP	0	0.0%	1	2.9%		
IUD	15	39.5%	18	52.9%	5.778	0.144
Other (Injection)	5	13.2%	1	2.9%		
**Family history of Cervical cancer**						
No	38	95.0%	37	92.5%		
Yes	2	5.0%	3	7.5%	0.455	0.500

χ^2^: Chi-square test; MC: Monte Carlo t: Student *t*-test.

**Table 5:** Distribution of the studied women according to their knowledge regarding cervical cancer (N = 80).

**Knowledge Regarding Cervical Cancer**	**Pre Intervention**				**Post Intervention**				
	**Study** **(n = 40)**		**Control** **(n = 40)**		**Study** **(n = 40)**		**Control** **(n = 40)**		
	**Freq.**	**%**	**Freq.**	**%**	**Freq.**	**%**	**Freq.**	**%**	
**Poor**	25	62.5%	19	47.5%	0	0.0%	15	37.5%	
**Fair**	14	35.0%	19	47.5%	26	65.0%	23	57.5%	
**Good**	1	2.5%	2	5.0%	14	35.0%	2	5.0%	
χ^2^ **(^MC^p)**	1.972 (0.433)				24.184 * (<0.001 *)				
**Mean ± SD**	38.71 ± 10.41		36.89 ± 16.33		66.25 ± 8.95		42.09 ± 15.95		
**t (p)**	0.593 (0.555)				8.355 * (<0.001 *)				
χ^2^: Chi-square test; MC: Monte Carlo t: Student *t*-test; * significant at *p*-value < 0.05.									
**Knowledge Regarding Cervical Cancer**	**Study** **(n = 40)**	**Control** **(n = 40)**	**t (p)**	**Interaction of Groups**	**Interaction of Time**	**Interaction Groups × Time**	**Effect Size (Cohen’s d)**	**95% CI Effect Size (Cohen’s d)**	
				**F/p**	**F/p**	**F/p**		**LL**	**UL**
Pre-Intervention	38.71 ± 10.41	36.89 ± 16.33	0.593 (0.555)	38.032 * (<0.001 *)	60.420 * (<0.001 *)	28.147 * (<0.001 *)			
Post-Intervention	66.25 ± 8.95	42.09 ± 15.95	8.355 * (<0.001 *)				1.515	1.125	2.611
**Δ (change score)**	27.5 ± 7.56	5.2 ± 5.50	U = 41.0 *, *p* < 0.001 *						
**95% CI (LL-UL)**	25.12–29.97	3.44–6.96							

F: F for ANCOVA test for the model; CI: Confidence interval; LL: Lower limit; UL: Upper Limit; *: Statistically significant at *p* ≤ 0.05.

**Table 6:** Distribution of the studied women according to their level of anxiety using the Beck Anxiety Inventory (BAI) (N = 80).

**Level of Anxiety Based on BAI**	**Pre Intervention**				**Post Intervention**				
	**Study** **(n = 40)**		**Control** **(n = 40)**		**Study** **(n = 40)**		**Control** **(n = 40)**		
	**Freq.**	**%**	**Freq.**	**%**	**Freq.**	**%**	**Freq.**	**%**	
Low	1	2.5%	3	7.5%	25	62.5%	5	12.5%	
Moderate	28	70.0%	24	60.0%	15	37.5%	26	65.0%	
High	11	27.5%	13	32.5%	0	0.0%	9	22.5%	
**χ^2^ (^MC^p)**	1.421 (0.512)				26.750 * (<0.001 *)				
**Mean ± SD**	38.68 ± 6.32		39.08 ± 11.84		22.20 ± 3.91		34.28 ± 9.53		
**t (p)**	0.189 (0.851)				7.414 * (<0.001 *)				
χ^2^: Chi-square test; MC: Monte Carlo t: Student *t*-test; * significant at *p*-value < 0.05.									
**Level of Stress Based on BAI**	**Study** **(n = 40)**	**Control** **(n = 40)**	**t (p)**	**Interaction of Groups**	**Interaction of Time**	**Interaction Groups × Time**	**Effect Size (Cohen’s d)**	**95% CI Effect Size (Cohen’s d)**	
				**F/p**	**F/p**	**F/p**		**LL**	**UL**
Pre-Intervention	38.68 ± 6.32	39.08 ± 11.84	0.189 (0.851)	21.750 * (<0.001 *)	63.259 * (<0.001 *)	19.050 * (<0.001 *)			
Post-Intervention	22.20 ± 3.91	34.28 ± 9.53	7.414 * (<0.001 *)				1.268	0.940	2.377
**Δ (change score)**	−16.5 ± 4.84	−4.80 ± 4.79	U = 72.50 *, *p* < 0.001 *						
**95% CI (LL-UL)**	−14.9–−26.0	−3.27–−13.0							

F: F for ANCOVA test for the model; CI: Confidence interval; LL: Lower limit; UL: Upper Limit; *: Statistically significant at *p* ≤ 0.05.

## Data Availability

The data presented in this study are available on request from the corresponding author due to privacy and confidentiality concerns, and ethical guidelines restricting data distribution.

## Supplementary Materials

The following supporting information can be downloaded at: https://www.mdpi.com/article/10.3390/healthcare14172684/s1, Table S1. Domains and representative sample items of the cervical cancer knowledge questionnaire.

## References

[1] Kimambo A. (2025). Cervical intraepithelial neoplasia: Histological classification and screening implications. J. Clin. Pathol. Lab. Med..

[2] World Health Organization (2025). Cervical Cancer.

[3] Dai W., Wang T., Chen L., Qiu Z., Chen P., Chen D. (2024). Immediate risk of cervical intraepithelial neoplasia and diagnostic value of colposcopy among cytology-negative women with oncogenic HPV: A retrospective study. BMC Women’s Health.

[4] International Agency for Research on Cancer (2026). Cervical Cancer Statistics.

[5] World Cancer Research Fund (2024). Cervical Cancer Statistics.

[6] ICO/IARC Information Centre on HPV and Cancer (2021). Egypt: Human Papillomavirus and Related Cancers, Fact Sheet.

[7] Centers for Disease Control and Prevention (2024). Cervical Cancer Risk Factors.

[8] Jhingran A., Meyer L.A., Gershenson D.M. (2022). Malignant diseases of the cervix: Microinvasive and invasive carcinoma: Diagnosis and management. Comprehensive Gynecology.

[9] National Comprehensive Cancer Network Cervical Cancer. https://www.nccn.org/guidelines/guidelines-detail?category=1&id=1426.

[10] American College of Obstetricians and Gynecologists (2024). Cervical Cancer Screening.

[11] Perkins R.B., Wentzensen N., Guido R.S., Schiffman M. (2023). Cervical cancer screening: A review. JAMA.

[12] Li C.C., Chang T.C., Huang C.H., Chang C.W., Tsai Y.F., Chen L. (2025). Impact of HPV test results and emotional responses on psychosocial burden among Taiwanese women: A cross-sectional study. BMC Women’s Health.

[13] Chamani S.S., Noroozieh M., Rezaee M. (2025). Integrating social-emotional learning and mindfulness in teacher education programs. J. New Trends Engl. Lang. Learn..

[14] Do T.-T., Giang T.-V. (2024). Mindfulness-based social-emotional learning program: Strengths and limitations in Vietnamese school-based mindfulness practice. Heliyon.

[15] Talvio M., Vuorinen J. (2026). Enhancing social and emotional learning: Insights from student teacher reflections in the UAE and Finland. Behav. Sci..

[16] Karakurt N., Durmaz H. (2025). The impact of mindfulness intervention on the subjective well-being of nursing students: An experimental study. BMC Psychol..

[17] Putri A.S., Ardianto H., Andriyaningsih A., Burmansah B. (2024). A study on mindful learning: Can mindfulness-based social-emotional learning strengthen student learning focus?. Int. J. Sci. Appl. Sci. Conf. Ser..

[18] Qin J., Liu Y., Wang Y., Li L., Hu X., Cao L., Zhou L., Zhou M. (2025). Mindfulness interventions in post-cervical cancer: Sexual, psychological, and quality of life impact. Psycho-Oncology.

[19] Kim J., Kim S., Kim M.W.D., Park Y.-H., Lee K., Chung D.S., Kim Y.H., Kweon Y.-S., Moon D.-S., Lee H.-Y. (2025). Effectiveness of the Mindfulness-Based Social–Emotional Growth (MSEG) Program in Enhancing Mental Health of Elementary School Students in Korea. Behav. Sci..

[20] Alvarado-García P.A.A., Soto-Vásquez M.R., Infantes Gomez F.M., Guzman Rodriguez N.M., Castro-Paniagua W.G. (2025). Effect of a mindfulness program on stress, anxiety, depression, sleep quality, social support, and life satisfaction: A quasi-experimental study in college students. Front. Psychol..

[21] Li Z., Lei D., Ting L., Yao R., Jing W., Na M. (2024). The impact of mindfulness intervention on negative emotions and quality of life in malignant tumor patients: A systematic review and meta-analysis. Front. Psychol..

[22] World Health Organization (2024). Immunizing Against HPV.

[23] Bruni L., Albero G., Serrano B., Mena M., Collado J., Gómez D. (2023). Human Papillomavirus and Related Diseases in China: Summary Report.

[24] Capili B., Anastasi J.K. (2024). An introduction to types of quasi-experimental designs. Am. J. Nurs..

[25] Cohen J. (1988). Statistical Power Analysis for the Behavioral Sciences.

[26] Harsha Kumar H., Tanya S. (2014). A study on knowledge and screening for cervical cancer among women in Mangalore city. Ann. Med. Health Sci. Res..

[27] Beck A.T., Epstein N., Brown G., Steer R.A. (1988). An inventory for measuring clinical anxiety: Psychometric properties. J. Consult. Clin. Psychol..

[28] Ibrahim M.B., Abdelreheem M.H. (2015). Prevalence of anxiety and depression among medical and pharmaceutical students in Alexandria University. Alex. J. Med..

[29] Albsoul-Younes A.M., Gharaibeh L., Younes D.N., Tahaineh L., Nuseir K.Q., Elsalem L., Alkayed Z., Wazaify M. (2026). Mental health among pharmacy students, assessing predictors of clinically significant anxiety and depression: A cross-sectional study from Jordan. BMC Med. Educ..

[30] Al-Issa I., Al Zubaidi A., Bakal D., Fung T.S. (2000). Beck Anxiety Inventory symptoms in Arab college students. Arab J. Psychiatry.

[31] An F., Dan X., An Y., Zhou L. (2020). Effects of mindfulness-based stress reduction on cervical cancer patients undergoing concurrent radiochemotherapy. Int. J. Clin. Exp. Med..

[32] Shawky F., Mohammed S., Wahed A., Attya A. (2024). Women’s perception regarding cervical cancer screening. Egypt. J. Health Care.

[33] Romli R., Shahabudin S., Saddki N., Mokhtar N. (2020). Effectiveness of a health education program to improve knowledge and attitude towards cervical cancer and Pap smear: A controlled community trial in Malaysia. Asian Pac. J. Cancer Prev..

[34] Debnath P., Pal J., Halder T., Ghosh M. (2024). Effect of health education on knowledge regarding cervical carcinoma among the female college students of Nadia district, West Bengal. Int. J. Res. Med. Sci..

[35] Okyere J., Ayebeng C., Dickson K.S. (2024). Early age at menarche and history of sexually transmitted infections significantly predict cervical cancer screening uptake among women aged 25–49 years: Evidence from the 2021 Côte d’Ivoire demographic and health survey. BMC Health Serv. Res..

[36] Crespo P., Herranz N., Estella V., Morencos E., Herranz M., Rodas G. (2025). Characteristics of the menstrual cycle and hormonal contraceptive use in elite Spanish basketball players. Front. Sports Act. Living.

[37] Fawzy A.M., Hossien D.Y.E.S., Ibrahim D.E.M., Taha D.R.R. (2023). Effect of educational program on knowledge and attitudes towards cervical cancer screening among women of reproductive age. Minia Sci. Nurs. J..

[38] Osman T.M.A., Keshk E.A., Mohamed O.M.O.I., Alomari H.A.S., Alshanini F.A.M., Alghamdi D.G.S.I., Alzahrani A.A.A., Alghamdi R.A. (2025). Knowledge, attitude, and practice toward cervical cancer and its screening among women from Al-Baha Area, Saudi Arabia. Int. J. Med. Dev. Ctries..

[39] Ikegwuonu S.N., Ewenyi E.O., Onuah I.A., Alumona F.C., Airaodion A.I. (2024). Awareness and prevalence of cervical cancer among women of reproductive age in Southeast, Nigeria. J. Cancer Manag. Res..

[40] Aldbyani A., Alhimaidi A., Chuanxia Z., Leng J., Alhadoor Z. (2025). Mindfulness and academic engagement among medical students: The roles of psychological capital and self-concept clarity. BMC Psychol..

[41] Mathkor D.M. (2025). Mindfulness and academic performance among nursing students in Saudi Arabia: A cross-sectional study. Sci. Rep..

[42] Inbar O.S., Tarrasch R. (2025). The effects of integrating mindfulness exercises into the elementary science curriculum: A cluster, randomized, controlled trial. Educ. Sci..

[43] Bordbar S., Ahmadinejad P., Bahmaei J., Yusefi A.R. (2024). The impact of mindfulness on academic achievement of students with the mediating role of adaptability: A structural equation modeling approach. BMC Med. Educ..

[44] Salem G.M.M., Hashimi W., El-Ashry A.M. (2025). Reflective mindfulness and emotional regulation training to enhance nursing students’ self-awareness, understanding, and regulation: A mixed-method randomized controlled trial. BMC Nurs..

[45] Bahri S., Montazeri S., Behrozi N., Maraghi E., Shahbazian H. (2022). The effect of mindfulness on quality of life and psychological well-being in women with cervical cancer. J. Adv. Pharm. Educ. Res..

[46] D’Souza F., Smyth L. (2025). The array of outcomes associated with mindfulness interventions in schools: A systematic review and meta-analysis. Mindfulness.

[47] Liu H., Cai K., Wang J., Zhang H. (2022). The effects of mindfulness-based interventions on anxiety, depression, stress, and mindfulness in menopausal women: A systematic review and meta-analysis. Front. Public Health.

[48] Kassie S.A. (2023). Prevention before intervention: Introducing mindfulness-based social-emotional learning in higher education institutions across the United Arab Emirates. Front. Educ..

