# Supplementary material for: Effect of Mindfulness-Based Social–Emotional Learning Approach on Knowledge and Anxiety Levels Regarding Cervical Cancer Prevention
Source: Healthcare (Basel). 2026 Aug 24;14(17):2684. doi: 10.3390/healthcare14172684 (PMC13565234; doi:10.3390/healthcare14172684)
Supplement: Supplementary file 1 [file healthcare-14-02684-s001.zip › Supplementary Table S1.pdf]

Supplementary Table S1. Domains and representative sample items of the cervical cancer knowledge questionnaire

Domain	Content assessed	Representative sample item
**Basic knowledge**	Definition and nature of cervical cancer	"What is cervical cancer?"
**Risk factors**	Factors associated with increased risk	"Which of the following is a risk factor for cervical cancer?"
**Causes/etiology**	HPV and other contributing factors	"Which infection is strongly associated with cervical cancer?"
**Signs and symptoms**	Common clinical manifestations	"Which of the following may be a warning sign of cervical cancer?"
**Screening**	Pap smear and HPV testing	"What is the main purpose of a Pap smear?"
**Prevention**	Preventive behaviors and screening	"Which measure can help prevent cervical cancer?"
**HPV vaccination**	Purpose and preventive role	"What is the main purpose of HPV vaccination?"
**Diagnosis**	Diagnostic procedures	"Which procedure is commonly used to investigate abnormal cervical findings?"
**Treatment**	Available treatment approaches	"Which treatment may be used for cervical cancer?"
**Complications**	Potential consequences of the disease	"Which of the following may occur if cervical cancer is not detected and treated?"
